# Angiotensin receptor neprilysin inhibitor in inotrope dependent heart failure patients: A case series

**DOI:** 10.34172/jcvtr.2020.53

**Published:** 2020-11-28

**Authors:** Sepideh Taghavi, Maryam Chenaghlou, Marzieh Mirtajaddini, Ahmad Amin, Nasim Naderi

**Affiliations:** ^1^Rajaie Cardiovascular Medical and Research Center, Tehran, Iran; ^2^Cardiovascular Research Center, Tabriz University of Medical Sciences, Tabriz, Iran; ^3^Cardiovascular Research Center, Kerman University of Medical Sciences, Kerman, Iran

**Keywords:** Heart Failure, Stage D, Angiotensin Receptor Neprilysin Inhibitor, Inotrope-Dependent

## Abstract

Patients with advanced heart failure (HF) symptoms constitute stage D heart failure with high mortality and less response to conventional guideline directed medical therapies. These patients are subjected to receive non-medical therapies including heart transplant or mechanical circulatory support for increasing survival. Considering the low availability and serious complications of these strategies,effective medical therapies for this group of patients would be pivotal for decreasing mortality and morbidity of them. Angiotensin receptor neprilysin inhibitor (ARNI) is a class of drugs approved for ambulatory heart failure patients. ARNI use like other groups of heart failure drugs has not been fully evaluated in end-stage heart failure patients. Herein, we describe four inotrope-dependent heart failure patients. Initiation of ARNI in these patients, lead to discontinuation of inotrope and reducing the need for inotrope in the follow-up period.

## Introduction

The main study population of heart failure (HF) clinical trials is the group with mild to moderate symptoms. Patients with advanced symptoms constitute low percentage in these studies and the evidence for the use of guideline directed medical therapy in this group of patients are less robust than other patients, besides these patients are at increased risk of HF medical treatment complications. Patients with advanced HFrEF (Heart Failure Reduced Ejection Fraction) are subjected to receive non-medical therapies such as heart transplantation, mechanical circulatory support and palliative care.^1^ Like other medical treatments for heart failure patients, the new category of drugs, angiotensin receptor neprilysin inhibitor (ARNI), has not been fully evaluated in end-stage HF patients, Herein, we describe four cases of HF patients with inotrope dependency state in their disease course. ARNI initiation leads to inotrope discontinuation.

## Case Presentation

### Case 1

He is a 56-year-old man with non-ischemic Dilated Cardiomyopathy (DCM) since 6 years ago and on guideline directed medical therapy (GDMT). Due to symptomatic heart failure (NYHA FC III) despite tolerated dose of GDMT and wide QRS (LBBB pattern), cardiac resynchronization therapy with defibrillator (CRT-D) was implanted according to Heart failure guidelines. Two years later, he was admitted with palpitation. atrioventricular (AV) node ablation and CRT-D mode changing to VVI (Ventricular pacing and sensing) mode was done due to supraventricular tachycardia episodes. There were another three admissions in six months later due to ventricular fibrillation (VF) episodes and implantable cardioverter defibrillation (ICD) shocks. Mexiletine was initiated. Two years ago, he had 9 admissions during ten months with dyspnea and decompensated heart failure despite optimal target dose of GDMT. Furosemide and milrinone were administered in each episode. Meanwhile, Sacubitril/valsartan (LCZ696) 24/26 mg twice daily was initiated with close follow-up regarding drug side effects. Due to acceptable the drug tolerance of patient without side effects like hypotension or worsening renal function, the drug was up-titrated to maximum tolerated dose. There is not any hospitalization due to de-compensation in recent fifteen months.

### Case 2

The patient is a 30-year-old man with DCM diagnosis since 4 years ago. ICD-VR (ICD single chamber) was implanted through the disease course. While he was on tolerated doses of HF medical therapies, the patient had three admissions with nausea, vomiting, epigastric pain and diarrhea. Upper GI endoscopy revealed, erythematous gastritis and erosive duodenitis. During these hospitalizations, inotrope was administered, due to hypotension and cardiogenic shock. Two weeks after the last hospitalization, he came with heart failure symptoms and in Interagency Registry for Mechanically Assisted Circulatory Support (INTERMACS) 3 state Profile. Decreased urine output was the prominent feature. Norepinephrine was initiated. Several trials for inotrope discontinuation were unsuccessful. Finally, we decided to initiate ARNI sacubitril/valsartan (S/V) 24/26 mg twice daily, at this time the patient systolic blood pressure was about 100 mm Hg. Interestingly, we could taper and off norepinephrine infusion. The patient did not show any adverse side effects of the drug and up-titration was done at follow-up visits. There was not any admission thereafter during 18 months.

### Case 3

He is a 35-year-old man with DCM diagnosis and on heart failure treatment since 3 years ago. ICD-VR was also implanted. There were three hospitalizations due to heart failure de-compensation during the second year of diagnosis despite administration of optimal tolerable dose of GDMT. In third admission, Norepinephrine was administered, due to hypotension and cardiogenic shock. Few days later, blood pressure increased to acceptable range, and we could withdraw norepinephrine. Elevated bilirubin level and reduced urine output developed after norepinephrine discontinuation. At that time, he was in INTERMACS 3 state. Milrinone was initiated, and he was doing well while on this inotrope but with reduction of it, there were the same signs of organ hypo-perfusion. We initiated S/V 24/26 mg twice daily when the patient systolic pressure was in acceptable range (~100 mm Hg). Few days later we could discontinue milrinone without organ damage signs and the patient discharged in good condition without any side effects of S/V. There was not any admission for heart failure during 1 year follow-up except one admission only two weeks after initiation of ARNI.

### Case 4

The patient is a-24-year old male with DCM diagnosis from one year ago. At his first admission with de-compensation symptoms while he was on optimal tolerable dose of GDMT two inotropes including norepinephrine and milrinone were administered. Inotropes could not be discontinued due to deterioration of congestive symptoms and low output state. S/V (24/26 mg twice daily) was initiated for patient after achievement of systolic blood pressure to the range of approximately 100 mm Hg. Few days later, besides improvement of patient condition without S/V side effects, inotropes discontinued and patient discharged in good condition. In about one year after that time, the patient was in acceptable function without any hospitalization for heart failure.

## Discussion

Beneficial effects of S/V has been shown in various disorders including, chronic HFrEF, acute HFrEF, HF with preserved ejection fraction, acute coronary syndrome, left ventricular remodeling, functional mitral regurgitation, hypertension, chronic kidney disease, pulmonary hypertension, cognitive function, obstructive sleep apnea, right ventricle dysfunction and reverse myocardial remodeling even in early stages of treatment. Extensive effects of S/V is probably due to diverse properties of natriuretic peptides including vasodilation, natriuresis, antiproliferative effects, modulation of renin-angiotensin-aldosterone system and vascular remodeling.^2,3,4^

The safety and efficacy of S/V in the setting of acute heart failure were evaluated in the multicenter, double-blind PIONEER trial. The higher time-average change from baseline to weeks 4 and 8 in plasma levels of NT-proBNP in the S/V group vs enalapril group was in favor of higher efficacy of S/V. Similar rates of worsening renal function or hyperkalemia between two groups were indicative of acceptable safety outcome of S/V. In this trial only NT-proBNP was used as a surrogate of S/V efficacy and cardiovascular outcomes were not evaluated.^5,6^

Our patients had acceptable renal function before initiation of S/V and we did not encounter significant complications of S/V including hypotension or worsening renal function after initiation of the drug and during the follow-up period.

In a recent report, S/V was initiated for five inotrope-dependent heart failure patients while they had inactive status on heart transplant waiting list due to significant pre-capillary pulmonary hypertension. Improvement in filling pressures, PA systolic pressure and pulmonary vascular resistance were observed following initiation of S/V leading to reactivation of them on heart transplant waiting list. The mechanism of this observation is proposed to be other than a simple vasodilatation. Right ventricular-PA (Pulmonary Artery) coupling improvement as the result of increased pulsatility index and decreased Pulmonary Vascular resistance (PVR) besides increase in cardiac index are the suggested mechanisms for this observation.^7^

Martyn et al in their study showed that patients with reduced cardiac output could be safely bridged from intravenous vasoactive therapy to S/V with maintenance of hemodynamic improvement including Pulmonary Artery Pulsatility index (PAPi) obtained from vasoactive drugs.^8^

Unfortunately we could not perform hemodynamic assessment immediately before and after initiation of S/V in our patients but it seems the same findings of the two mentioned studies have been occurred in our patients leading to clinically improvement in condition of the patients.

Although these recent studies and our report have been done on small sample sizes, but it might be an indicator of this drug potential for use in stage D and inotrope dependent HF patients.

## Conclusion

Limited available treatments for stage D HF patients necessitate new treatment strategies in this group. Promising reported results of ARNI use in stage D heart failure patients could represent this class of drugs as a potential treatment in end stage HF patients.

## Competing interest

The authors declare that there is no conflict of interest.

## Ethical approval

This study was approved by the ethics committee of Rajaie Cardiovascular, Medical and Research Center. Written informed consent was obtained from patients.
